# Boron-assisted abiotic polypeptide synthesis

**DOI:** 10.1038/s42004-023-00885-7

**Published:** 2023-05-11

**Authors:** Yuki Sumie, Keiichiro Sato, Takeshi Kakegawa, Yoshihiro Furukawa

**Affiliations:** Department of Earth Science, Tohoku University, 6-3, Aza-aoba, Aramaki, Aoba-ku, Sendai, 980-8578 Japan

**Keywords:** Origin of life, Geochemistry, Peptides, Organocatalysis

## Abstract

The emergence of proteins and their interactions with RNAs were a key step in the origin and early evolution of life. The abiotic synthesis of peptides has been limited in short amino acid length and is favored in highly alkaline evaporitic conditions in which RNAs are unstable. This environment is also inconsistent with estimated Hadean Earth. Prebiotic environments rich in boron are reportedly ideal for abiotic RNA synthesis. However, the effects of boron on amino acid polymerization are unclear. We report that boric acid enables the polymerization of amino acids at acidic and near-neutral pH levels based on simple heating experiments of amino acid solutions containing borate/boric acid at various pH levels. Our study provides evidence for the boron-assisted synthesis of polypeptides in prebiotically plausible environments, where the same conditions would allow for the formation of RNAs and interactions of primordial proteins and RNAs that could be inherited by RNA-dependent protein synthesis during the evolution of life.

## Abstract (web summary)

Prebiotic environments rich in boron have been postulated to be ideal for abiotic RNA synthesis, but the effects of boron on amino acid polymerization are unclear. Here, boric acid is shown to enable the polymerization of amino acids at acidic and near-neutral pH levels.

## Introduction

RNAs are biopolymers that can carry genetic information and catalyze biological reactions^1^. Interactions between proteins and RNAs support essential biological processes, including transcription, translation, and regulation of gene expression^2^. The origin of these interactions was a key step in the origin of life and is thought to have occurred at the beginning of the RNA world or the transition from the RNA world to DNA–protein systems^2–8^.

Various proteinogenic amino acids would have been generated by terrestrial synthesis and extraterrestrial delivery to prebiotic Earth^9,10^. Prebiotic peptide synthesis has been investigated for decades in different geological settings, including volcanic geothermal fields, hydrothermal fields, sea-floor sediments, and tidal flats^11–16^. The effects of minerals, salts, ions, and pH have also been investigated^11–14^. Under highly alkaline conditions, peptide synthesis was favored, but the synthesized peptides were limited in terms of 20-mer oligopeptides (Gly_20_), unless the amino acids were chemically activated^8^. Highly alkaline conditions are not compatible with RNA synthesis due to its low stability. Thus, the origins of these essential biopolymers have been regarded separately. Another approach of the prebiotic peptide synthesis is the multistep coupling of an aminonitrile with thioacetic acid, although the presence of thioacetic acid on prebiotic Earth has not been clear, and the products are limited in Gly_6_^17^.

Previous studies found that borate can bind ribose and selectively stabilize it among aldopentoses, e.g., ^18–20^. Boric acid is important prebiotic reagent for regioselective phosphorylation of ribose and nucleosides to form ribonucleoteds^21–25^. Furthermore, boric acid can fix ribose in furanose in ribonucleoside synthesis^23^. Therefore, environments rich in borate and boric acid were potentially ideal for the formation of RNA on prebiotic Earth^26^. A potential effect of borate on prebiotic peptide formation was previously discussed^27^, but it remains unclear. In this study, we report the effect of boric acid in the polymerization of Gly. This effect was substantial under acidic and neutral evaporative conditions, and thus boric acid could promote prebiotic peptide synthesis in the same environment where RNAs are stable.

## Results and discussion

### Formation of polypeptides and the effects of boron

**Fig. 1:**
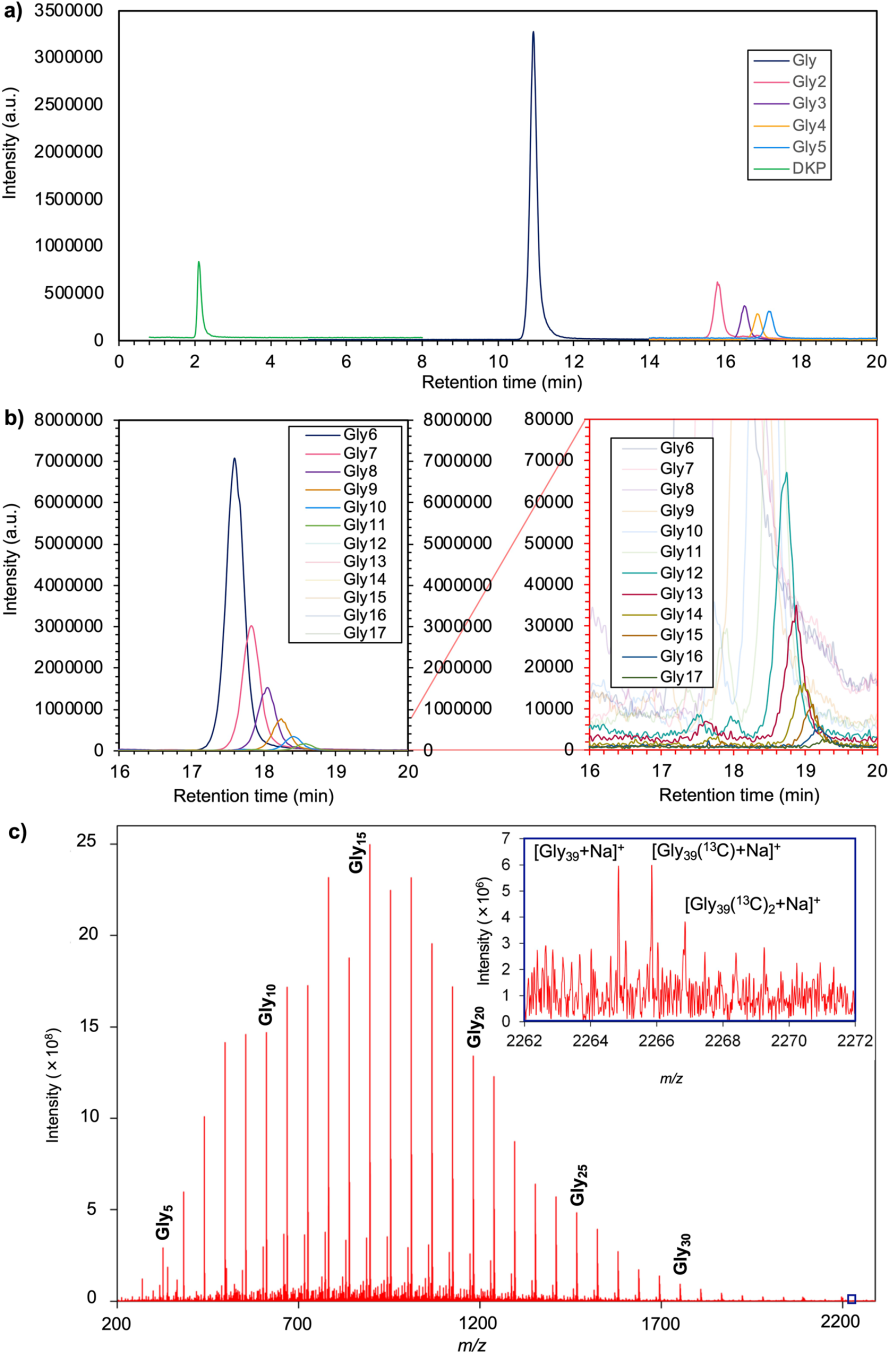
Identification of Gly peptides formed in the evaporation experiment. **a** Liquid chromatography–mass spectrometry (LC–MS) analysis of Gly_1–6_. **b** LC–MS analysis of Gly_6–17_. Note that a diluted sample of (**b**) was analyzed in (**a**). Gly_1–6_ were identified and quantified according to mass and LC–MS retention time using commercially available standards. Gly_7–17_ were identified by an increasing trend in LC–MS retention time. Detection of the product peptides was limited to short oligomers due to the low solubilities of long oligomers. **c** Fourier transform ion cyclotron resonance mass spectrometry (FTICR-MS) analysis of the product peptides. Both [M+Na]^+^ and [M+H]^+^ ions were detected for many peptides.

**Fig. 2:**
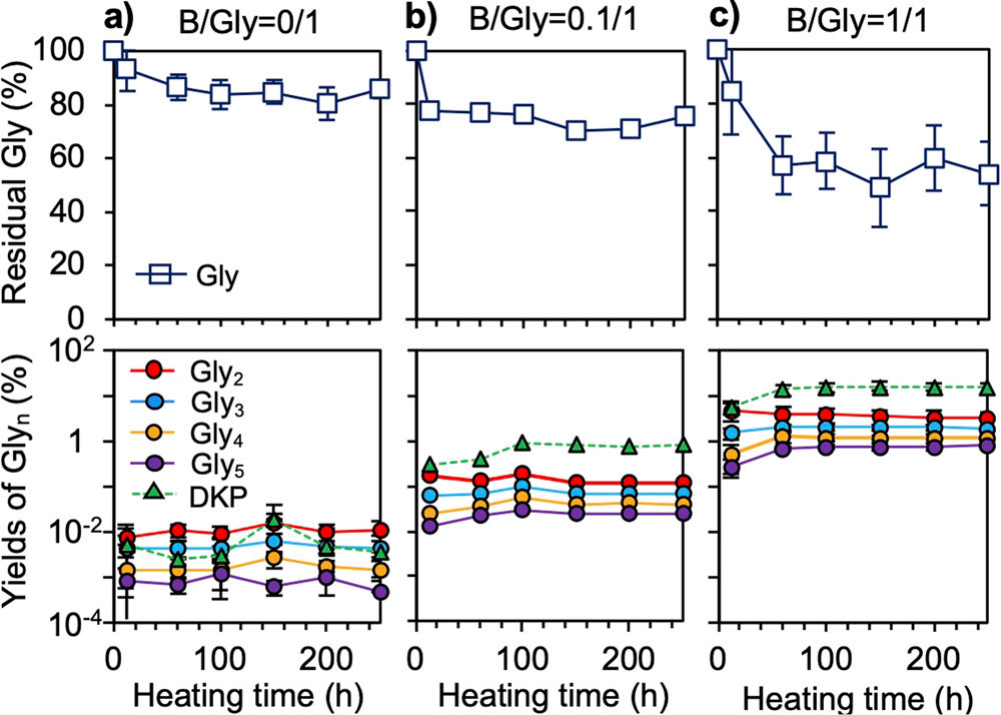
Effects of the boric acid and Gly molar ratio on peptide synthesis at pH 6 and 130 °C. **a** The molar ratio of Gly/Boron = 1/0. **b** The molar ratio of Gly/Boron = 1/0.1. **c** The molar ratio of Gly/Boron = 1/1. Error bars representing standard deviation (±1σ) are provided for (**a**) and (**c**) (*n* = 3). The following indicators are used, dark blue line: residual glycine, red line: Gly_2_, light blue line: Gly_3_, yellow line: Gly_4_, purple line: Gly_5_, and green line: DKP.

**Fig. 3:**
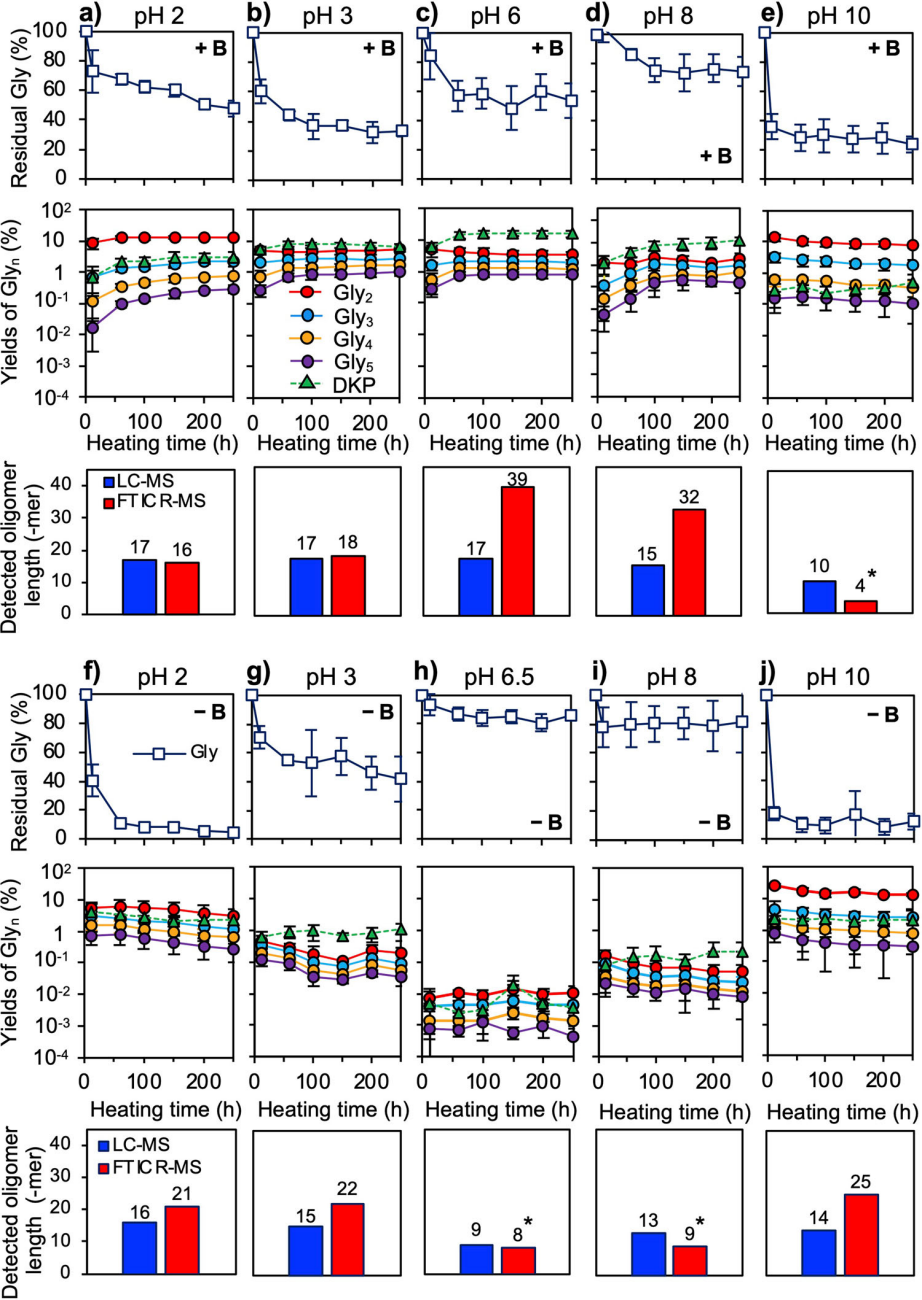
Effects of pH on peptide yield and residual Gly (B/Gly = 1) at 130 °C. **a** Yields of short peptides (Gly_2–5_ and DKP), residual amounts of glycine, and the peptides detected by LC–MS and FTICR-MS from a B-containing solution at pH 2, **b** pH 3, **c** pH 6, **d** pH 8, and **e** pH 10. **f** Products from a B-free solution at pH 2, **g** pH 3, **h** pH 6.5, **i** pH 8, and **j** pH 10. The following indicators are used, dark blue line: residual glycine, red line: Gly_2_, light blue line: Gly_3_, yellow line: Gly_4_, purple line: Gly_5_, and green line: DKP. Highly soluble and less soluble products were detected by LC–MS and FTICR-MS, respectively. Error bars represent standard deviation (±1σ; *n* = 3). *Peptides detected in the first extraction solution due to the absence of less soluble products.

Here, we report the abiotic polymerization of amino acids (up to Gly_39_) by simple thermal evaporation of a near-neutral amino acid solution (pH 6 and 8) containing boric acid at 130 °C for 200 h (Fig. 1, Supplementary Figs. 1–3 and Supplementary Tables 1 and 2). The yields of short Gly peptides and the lengths of detectable long peptides were dependent on the amount of boric acid in the starting material (Fig. 2). In the absence of boric acid, the longest product peptides were Gly_9_ and Gly_13_ under the same pH and temperature. The total yield of short oligomers (i.e., Gly_2–5_ and diketopiperazine [DKP]) was limited to 0.00025 times that of the thermal evaporation experiment with boric acid at pH 6 (Fig. 3). The yields of short oligomers were comparable under different pH conditions in the presence of boron species, but the formation of long oligomers was the most substantial under near-neutral conditions (pH 6–8) at 130 °C (Fig. 3a–e). In contrast, in the absence of boron species, both the yields and lengths of oligomers were higher under acidic and alkaline conditions than under neutral conditions (Fig. 3f–j). The catalytic effects of boron species on peptide synthesis were also shown under an acidic condition at 90 °C (Supplementary Fig. 4), whereas this effect was not shown under the acidic condition at 130 °C. Substantial conversion of Gly to a black by-product under the high-temperature acidic condition would have overwhelmed the boron-assisted peptide synthesis at 130 °C, while this by-product synthesis was negligible at 90 °C (Supplementary Figs. 5 and 6). Wet-dry cycles did not provide significant effects on the yields and the length of product Gly peptides (Supplementary Fig. 7). The enhancement of peptide synthesis by boric acid was also confirmed for alanine, although longer reaction durations will be required for the formation of polyalanine (Supplementary Fig. 8).

**Fig. 4:**
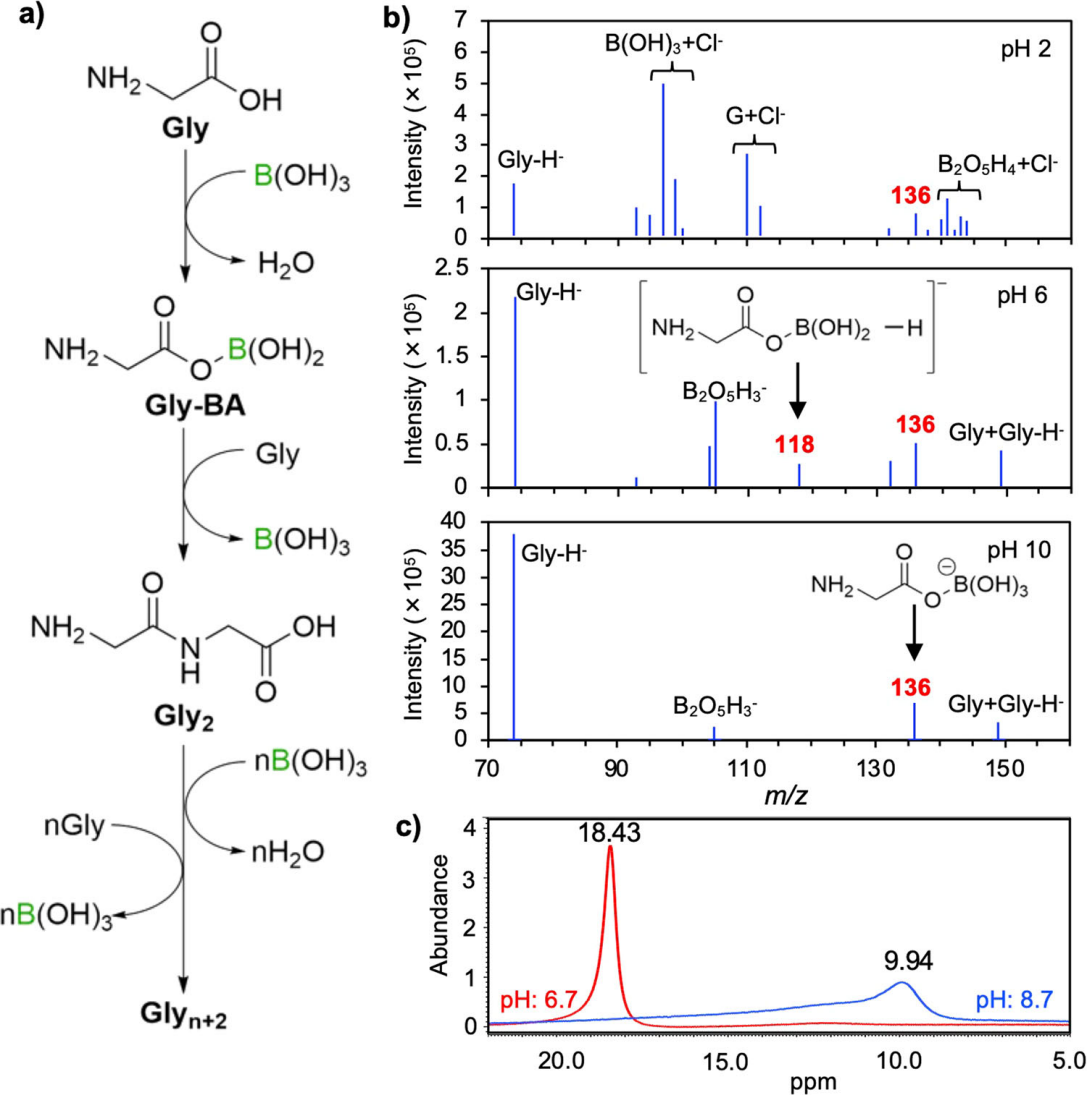
Formation of peptide bonds through ester synthesis between Gly and boric acid. **a** Reactions of Gly polymerization assisted by boric acid. **b** Negative ESI–MS spectra showing the formation of Gly-BA and Gly-B in solutions at pH 6 and 10. **c** ^11^B-NMR spectra of borate/boric acid solutions containing Gly at a near-neutral and alkaline pH. The sharp peak at 18.43 ppm and broad peak at 9.94 ppm represent the speciation of B as boric acid and the mixture of borate and boric acid to a borate anion and Gly, respectively. Note that the signals from borate and its esters would be more effectively detected than boric acid and its esters because negative ESI–MS is more sensitive to anions.

Organic boron species and boric acid form borate esters with carboxylic acids and catalyze the formation of amide bonds with amines by refluxing their water-free organic solvents^28–30^. We investigated the formation of esters between Gly and borate (Gly-B) and Gly and boric acid (Gly-BA) in starting solutions of different pH. It was found that Gly and boron hydroxides formed Gly-BA in the near-neutral solution and Gly-B in the acidic, near-neutral, and alkaline solutions (Fig. 4a, b). ^11^B-nuclear magnetic resonance (NMR) of the starting solutions showed that the speciation of bulk boron hydroxides was typical in that borate and boric acid dominated at pH >9 and <9, respectively (Fig. 4c). However, the speciation of boron that formed esters with Gly differed from that of typical boron hydroxide solutions, which is consistent with the findings of a previous study^31^. It remains unclear whether Gly-B or Gly-BA is a more efficient reaction species for peptide synthesis. We also conducted FT-IR analysis of dried starting materials (Supplementary Fig. 7). A typical peak reported as an asymmetric ν_asym_(BO)/BO_4_ stretch of boric acid appearing in the 1190 cm^−1^ region was shifted to 1227 cm^−1^ region in the analysis of a precipitate of a solution containing Gly and boric acid, supporting the formation of an ester compound as investigated in a previous study^32^.

The reactions under highly acidic and alkaline conditions without boron converted 70% and consumed 90% of the initial Gly, generating brown to black by-products (Fig. 3j and Supplementary Figs. 7 and 8), while the reactions under neutral conditions with boron converted 50% and consumed 50% of the initial Gly (Fig. 3c). Thus, boron-assisted peptide synthesis is a highly effective reaction with limited formation of by-products. When the boron esters react with Gly to form a peptide bond, borate and boric acid are released as the same form of boron species (Fig. 4a). Thus, small amounts of boron species can continuously catalyze peptide synthesis.

### Implication to Hadean Earth

Amino acid oligomerization is favored under highly alkaline and acidic conditions^12,14,15^. Alkaline and acidic environments are common around submarine hydrothermal areas^33–35^. However, dehydration during the formation of a peptide bond does not effectively occur in water-rich environments, and RNAs are not stable in highly acidic and alkaline conditions. A recent finding suggested that neutral environments favored the polymerization of ribonucleotide to form primordial RNA^36^. The pH of the Hadean ocean is unclear, but the pH of the early Archean ocean is estimated to have been near-neutral (pH ~6.5–7)^37^. This pH is compatible with amino acid polymerization in the presence of boric acid, as shown in this study. Given the presence of small land environments on the Hadean Earth^38^, evaporitic basins would have been common. Boron occurs as a borosilicate mineral, tourmaline, in 3.8-Ga old metasediments of Isua Greenland^39^. Boron isotope compositions in the tourmaline suggest that dissolved borate and boric acid were enriched in isolated basins on early Archean Earth, and may date back to Hadean Earth^26,39^. These isolated basins would also have accumulated amino acids and other organic compounds compared with the open ocean. Thus, evaporitic environments enriched in amino acids and boric acid would have been ideal for the formation of peptides on Hadean Earth. The temperatures of these environments would have been lower than those in our experiments, but the catalytic effects of boron species to promote Gly oligomerization were evident even at a lower temperature, i.e., 90 °C (Supplementary Fig. 4). Small proteins—composed of a limited amount of amino acids (e.g., <100)—have various biological functions^40,41^. The length of the peptides formed in this study (i.e., 39 amino acid residues) is typical of small proteins. The catalytic effect of boric acid is evident for Gly and Ala, and thus it may catalyze the polymerization of multiple amino acids, although this is a subject of future investigation.

The near-neutral pH at which boron-assisted polypeptide synthesis occurs also favors the stability of RNA, unlike the highly alkaline and acidic experimental conditions of previous studies^12–15^. Given that boron species can promote abiotic RNA synthesis, it is likely that the same environments of prebiotic Earth allowed for the interaction of abiotic polypeptides and RNAs, which would have facilitated the formation of these complex polymers with various functions. Some riboswitches are amino acids, and they can be associated with translation. This suggests that RNA could have interacted under prebiotic environments with amino acids or small peptides^42^. Proto-peptides composed of several amino acids and hydroxy acids are known to increase the stability of RNAs^7^. The core domain of ancient RNA polymerase might have been composed of ∼40 amino acids^43^. Furthermore, some peptides can activate the functions of RNA polymerase ribozyme^6,44^. Therefore, evaporitic basins on Hadean Earth might have provided primordial functional polymers composed of peptides and RNAs that could be inherited by RNA-dependent protein synthesis during the evolution of life.

## Materials and methods

All experiments were conducted using glass vials placed in an aluminum block in an electric furnace. The size of the glass vials was 1.5 mL, 32 mm in height, 6 mm upper interior diameter, and 9.5 mm bottom interior diameter. The glass vials were baked at 500 °C for 6 h before use. Most of the reagents were purchased from FUJIFILM Wako Pure Chemical Corporation (Osaka, Japan). Ultrapure water was prepared using a Milli-Q Integral (18.2 MΩ·cm, <5 ppb TOC). The starting solution was prepared with 600 μL of Gly solution (0.5 mol L^−1^) added to 0.5 mol boric acid, and the pH was adjusted with NaOH or HCl as shown in Supplementary Table 3. When the pH was not adjusted, the pH of the starting solutions was 6.5 and 5.3 in the absence and presence of boric acid, respectively. In we-dry cycle experiments, 600 μL of water was added to the vial and vortexed at 100 and 200 h.

The product was dissolved in 300 μL water and the resulting peptides were quantified using an ultra-performance liquid chromatography–tandem mass spectrometer (LC–MS/MS; Shimadzu LCMS-8040) using a VC-50 2D column (2.0 × 150 mm, 5 μm; Shodex) set to 50 °C. Eluent composition was changed linearly from 70% to 37% acetonitrile over 17 min at a flow rate of 0.25 mL min^−1^. Peptides were analyzed in the positive mode of electrospray ionization (ESI) with the nebulizer gas flow set to 2.5 L min^−1^, drying gas flow to 10 L min^−1^, desolvation temperature to 250 °C, and heat block temperature to 400 °C. Commercially available standard peptides of Gly_2–5_ and DKP were used for quantification. For samples in which long oligomers and polymers were formed, residues were present after dissolution with the first extract in 300 μL water. These residues were extracted into a second solution with 10–100 μL water or water/methanol, and the solutions were analyzed with a matrix-assisted laser desorption/ionization (MALDI) Fourier transform ion cyclotron resonance mass spectrometer (FTICR-MS; solariX 9.4T; Bruker Daltonics, Billerica, MA, USA) or MALDI time of flight mass spectrometer (REFLEX III; Bruker Daltonics) with 2,5-dihydroxybenzoic acid as the matrix. Gly-B and Gly-BA formation was monitored with the negative mode of ESI with direct infusion of the diluted starting materials. The settings of the mass spectrometer were the same as for the LC–MS/MS analysis.

^11^B-NMR experiments were conducted using an 800 MHz NMR spectrometer (JNM-ECA800). Spectra were collected with 8–32 scans with a 2-s relaxation delay. The chemical shift values were reported based on a reference standard material (boric acid, pH 7, at 16.067 ppm). Samples were dissolved in D_2_O. FT-IR analysis of glycine and the mixture of glycine and boric acid was conducted with JASCO FT/IR-6300 with spectrum resolution of 4 cm^−1^ and 100 cumulative times.

## Supplementary information

Supplementary Information

## Data Availability

The datasets generated during and/or analyzed during the current study are available from the corresponding author on reasonable request.
