# Supplementary material for: Boron-assisted abiotic polypeptide synthesis
Source: Commun Chem. 2023 May 11;6:89. doi: 10.1038/s42004-023-00885-7 (PMC10175494; doi:10.1038/s42004-023-00885-7)
Supplement: Supplementary file 1 — Supplementary Information [file 42004_2023_885_MOESM1_ESM.pdf]

Supplementary Information for

Boron-assisted abiotic polypeptide synthesis

Yuki Sumie[1], Keiichiro Sato[1], Takeshi Kakegawa[1], Yoshihiro Furukawa[1]*

[1]Department of Earth Science, Tohoku University, 6-3, Aza-aoba, Aramaki, Aoba-ku, Sendai 980-8578, Japan.

*Corresponding author

E-mail address: furukawa@tohoku.ac.jp

Supplementary Fig. S1–S9

Supplementary Table S1–S3

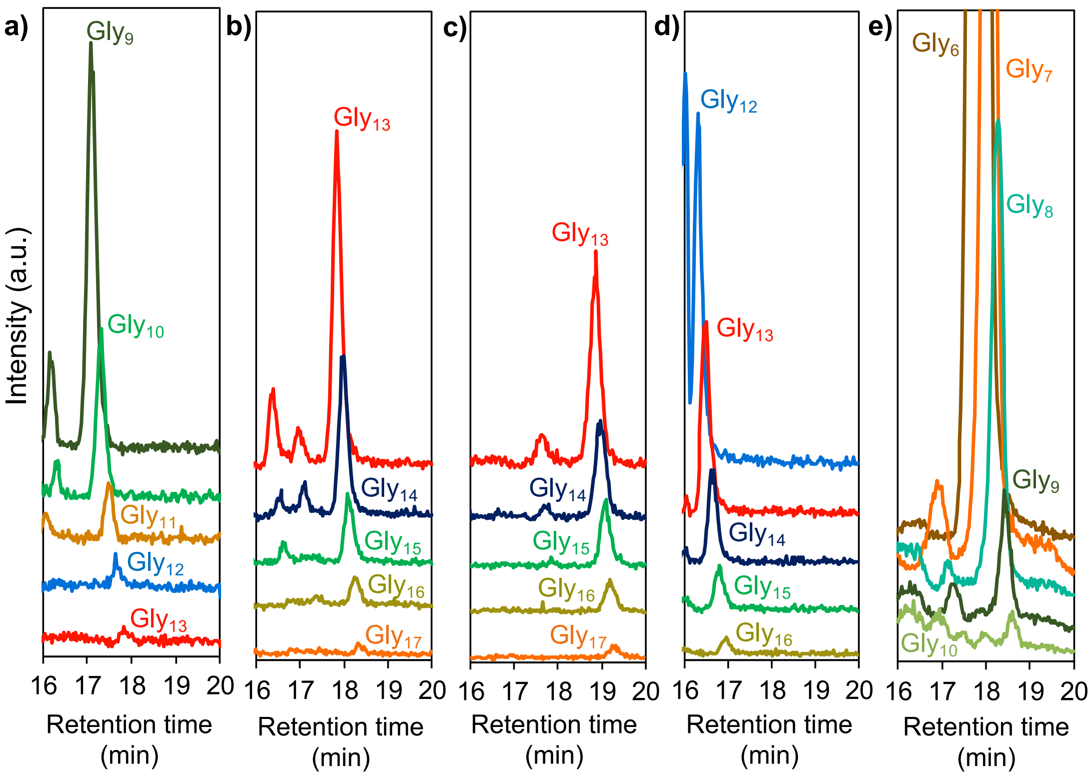

Fig. S1. LC–MS chromatograms of peptides produced in borate/boric acid solutions at 130 °C, 60h. a) Products after 250 h of heating at pH 2.0. **b)** 60 h of heating at pH 3.0. **c)** 100 h of heating at pH 6.1. **d)** 250 h of heating at pH 7.7. **e)** 250 h of heating at pH 10.

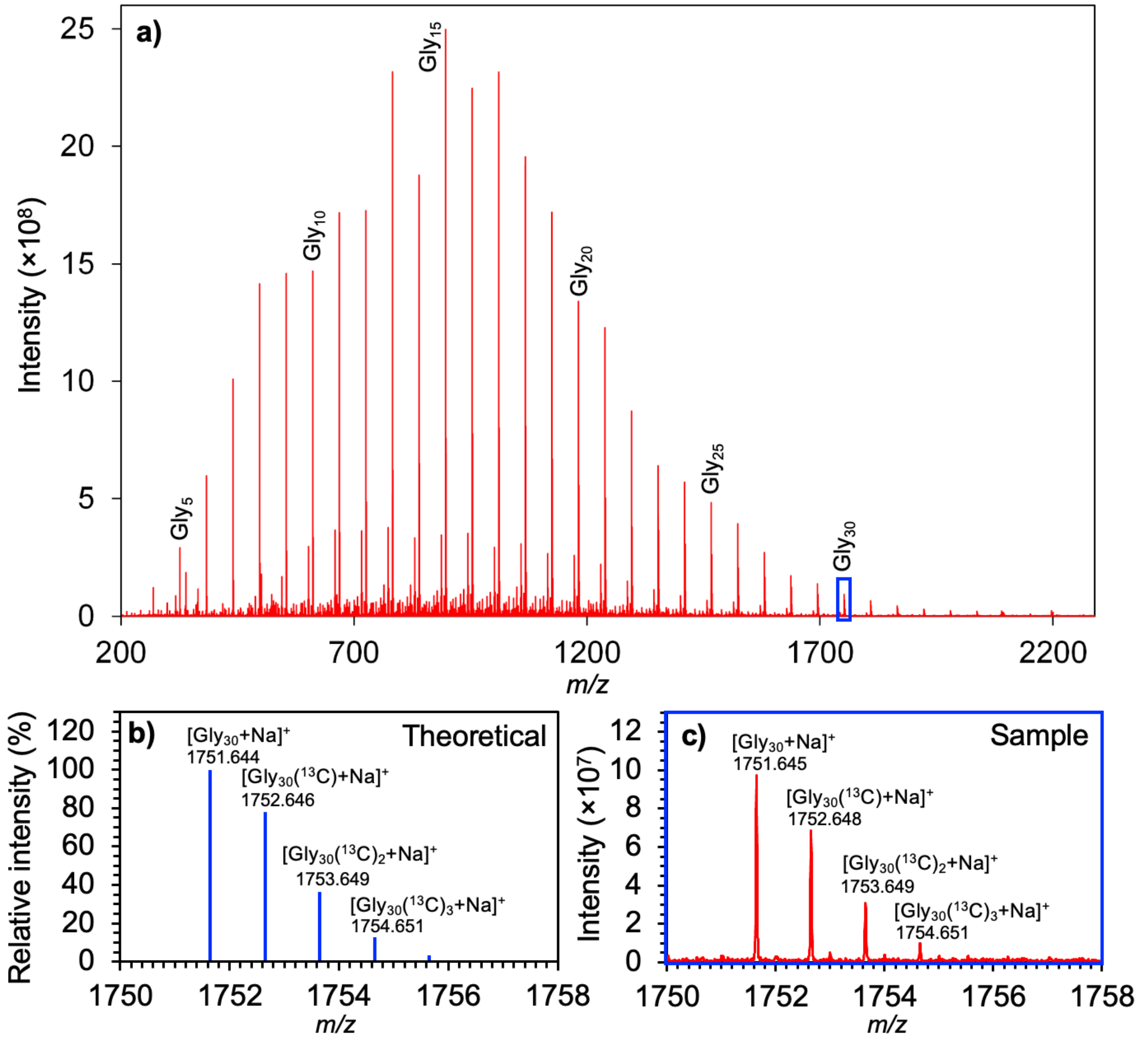

Fig. S2. Comparison of isotopomer abundances between products and theoretical peptides. **a)** Mass spectra of peptides produced in the boric acid solution at pH 6 and 130 °C. **b)** Theoretical m/z values and the abundances of Gly_{30}. **c)** Observed isotope m/z values and the abundances of Gly_{30}.

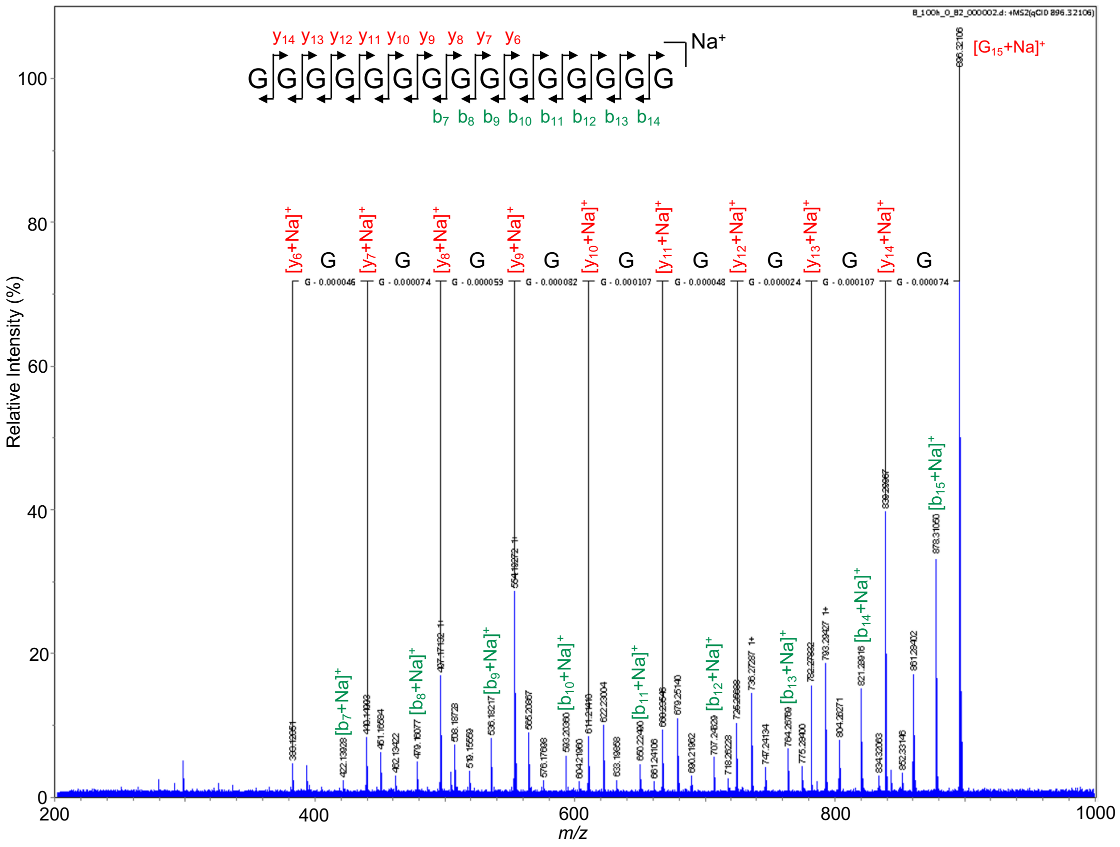

Fig. S3. Tandem MS fragmentation spectra of a detected peptide. The precursor ion was Gly_{15} in Fig. 1B. The fragments show that Gly_{15} is composed of repeating Gly sections.

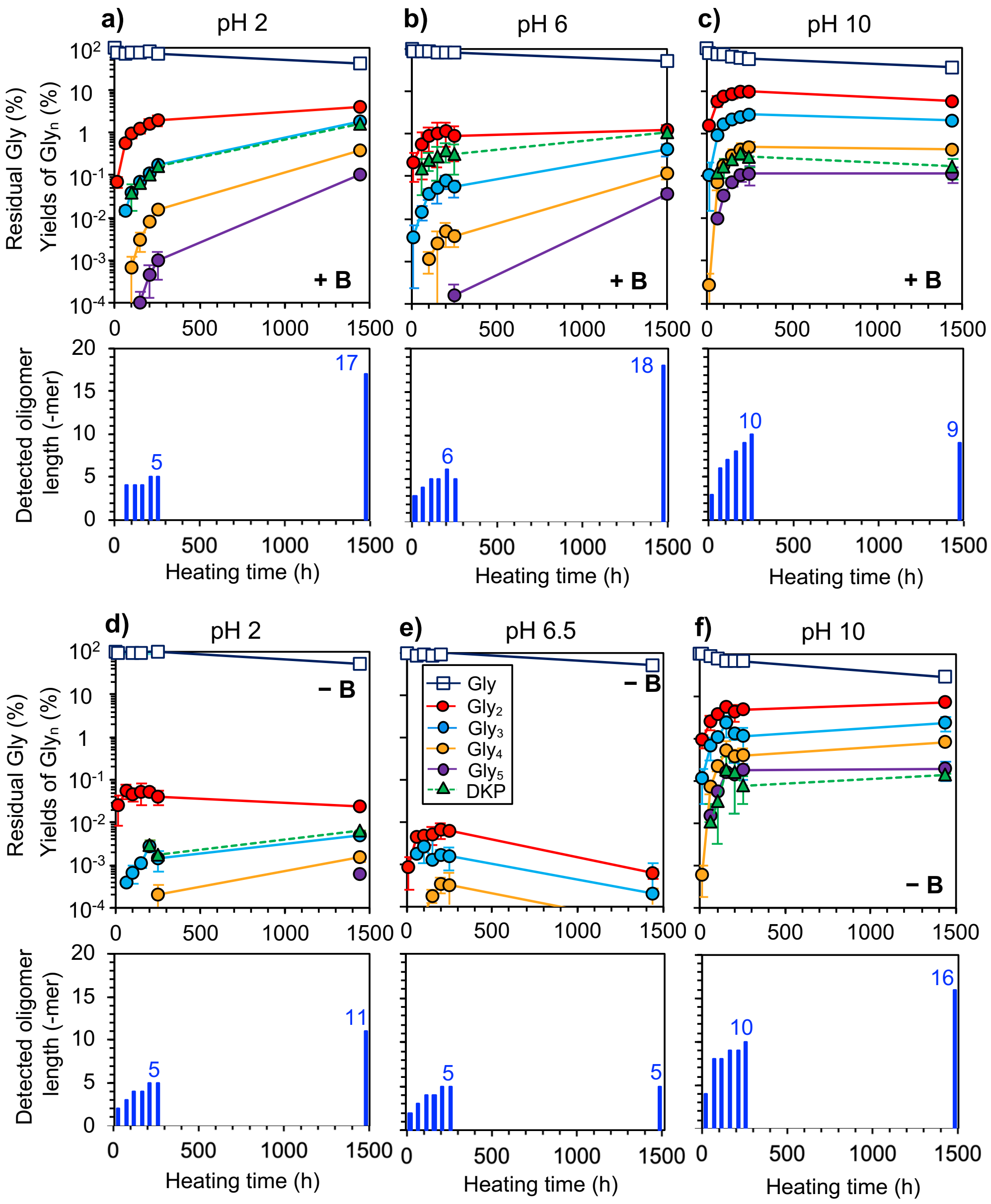

Fig. S4. Effects of initial pH on short peptide yields ($Gly_{2\text{-}5}$ and DKP), proportion of residual Gly, and detected peptide length at 90 °C. a) in a boron-absent solution at pH 2, **b)** pH 3, **c)** pH 6.5, **d)**, pH 8, and **e)**, pH 10. **f)**, Products from a B-containing solution at pH 2, **g)**, pH 3, **h)**, pH 6, **i)**, pH 8, and **j)**, pH 10. There were no insoluble products in the 300 μL of water. Thus, the peptides were analyzed by LC–MS. Error bars representing standard deviation (±1σ) are provided (n = 3). The following indicators are used, dark blue line: residual glycine, red line: Gly_2, light blue line: Gly_3, yellow line: Gly_4, purple line: Gly_5, and green line: DKP.

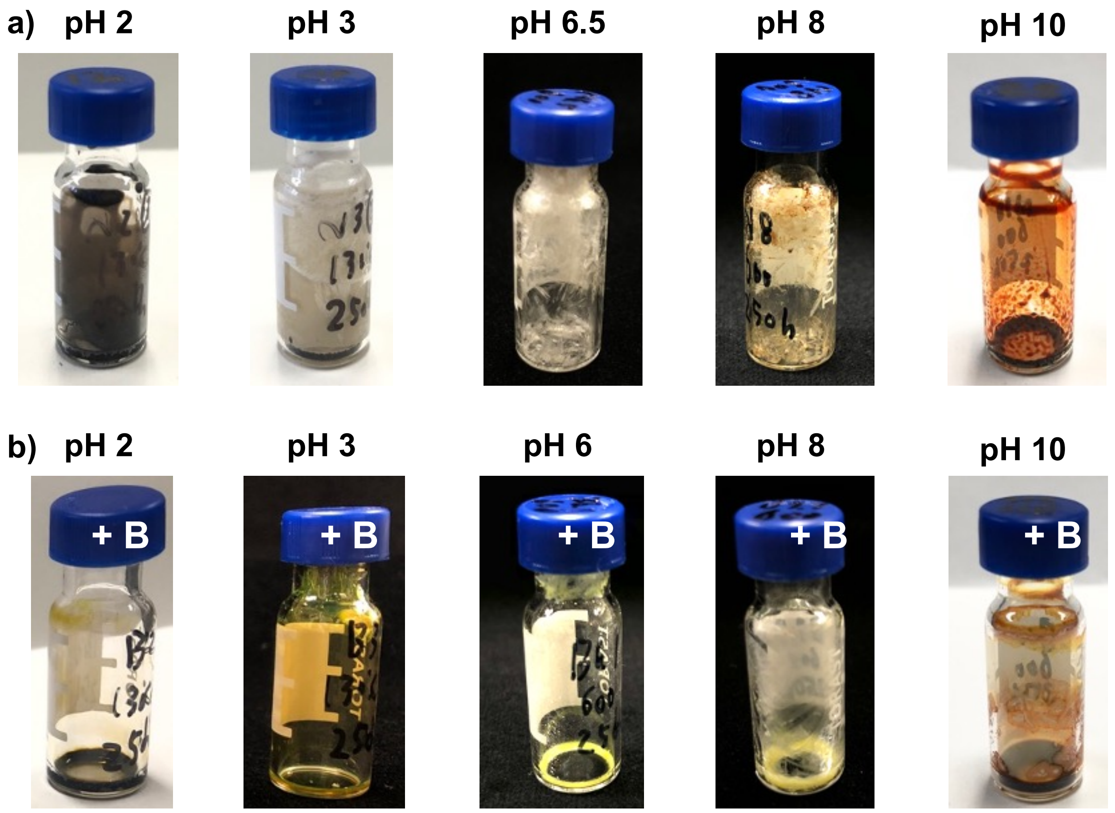

Fig. S5. Appearance of samples after thermal evaporation at 130 °C for 250 h. Experiments in the absence of boron **(a)** and the presence of boron **(b).** Products from highly acidic and alkaline solutions became black and brown, respectively.

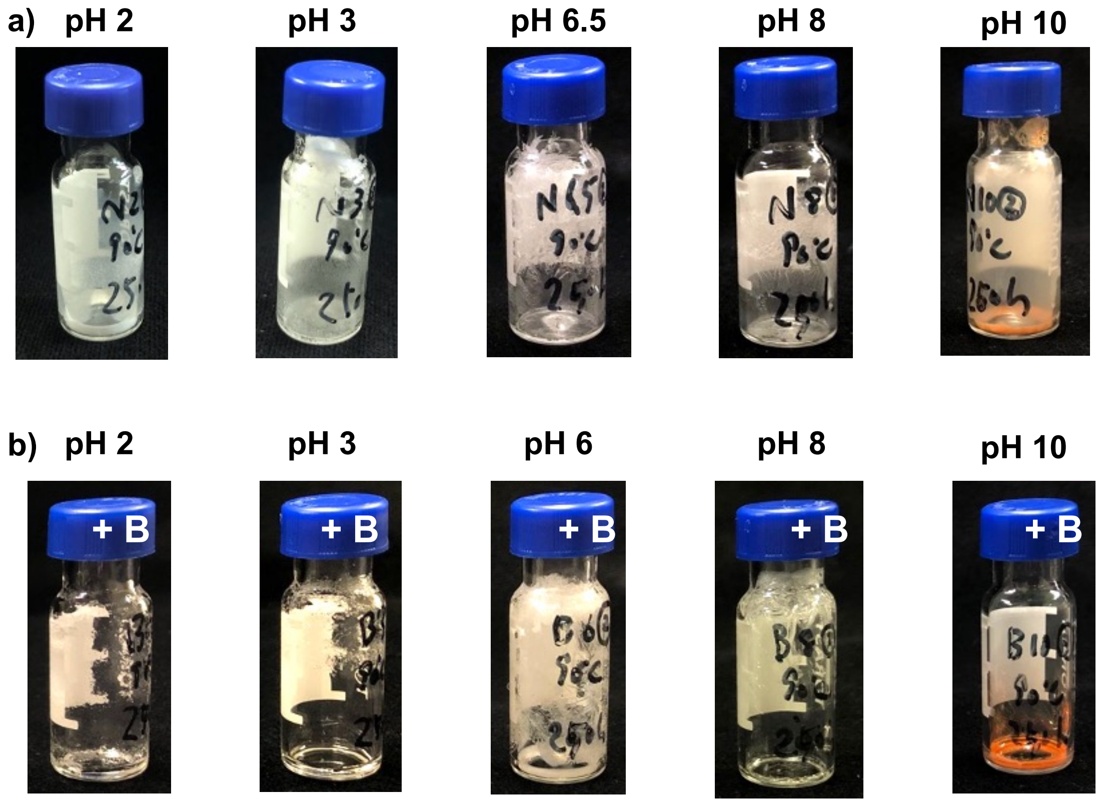

Fig. S6. Appearance of samples after thermal evaporation at 90 °C for 250 h. Experiments in the absence of boron **(a)** and the presence of boron **(b).** Products from highly alkaline solutions became brown.

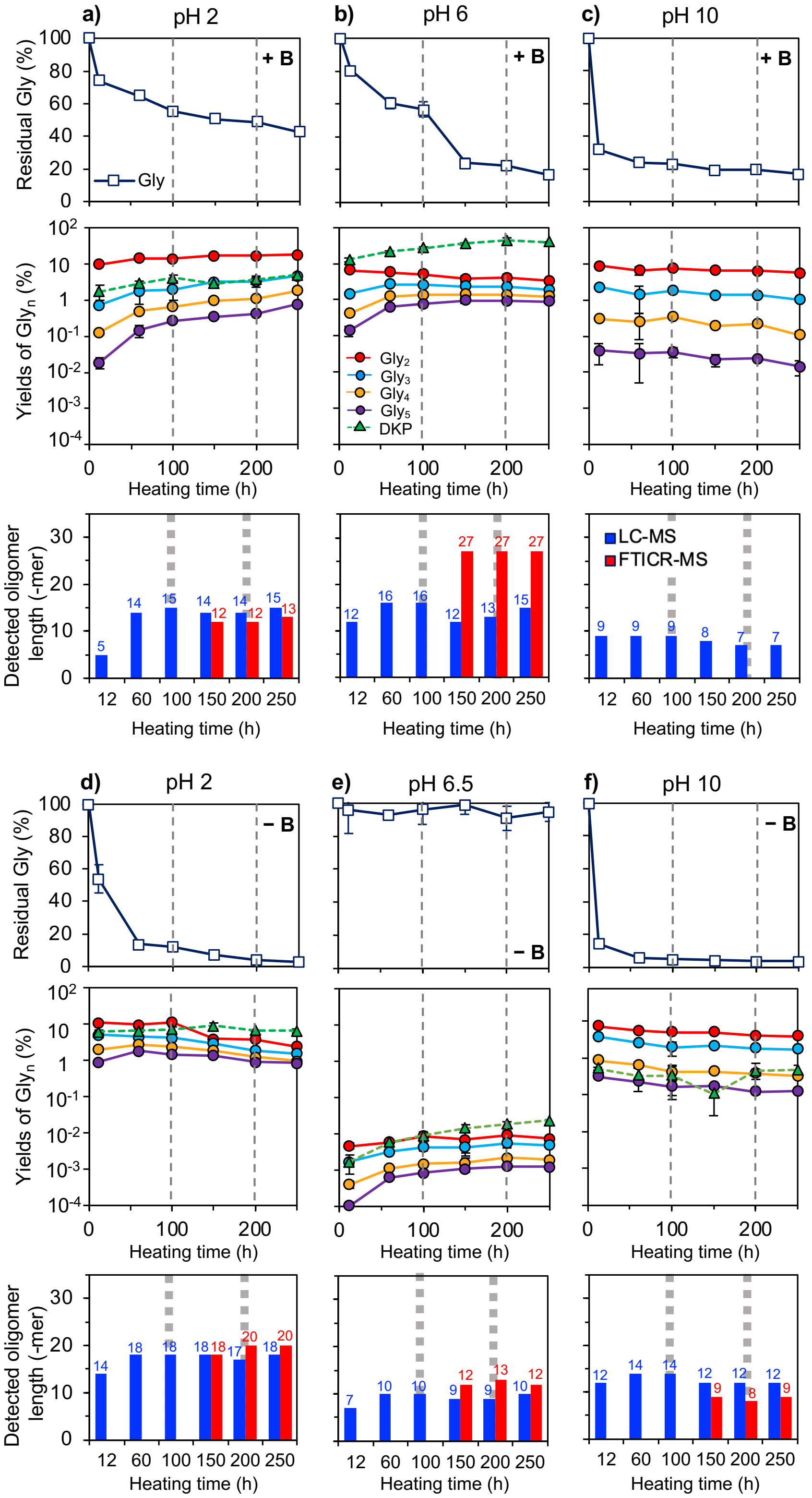

Fig. S7. Effects of wet-dry cycles on the yields and length of product peptides and residual amounts of Gly at 130°C. Incubations in the presence of equivalent molar boron at **a)** pH 2, **b)** pH 6, and **c)** pH 10. Incubations in the absence of boron at **d)** pH 2, **e)** pH 6, and **f)** pH 10.

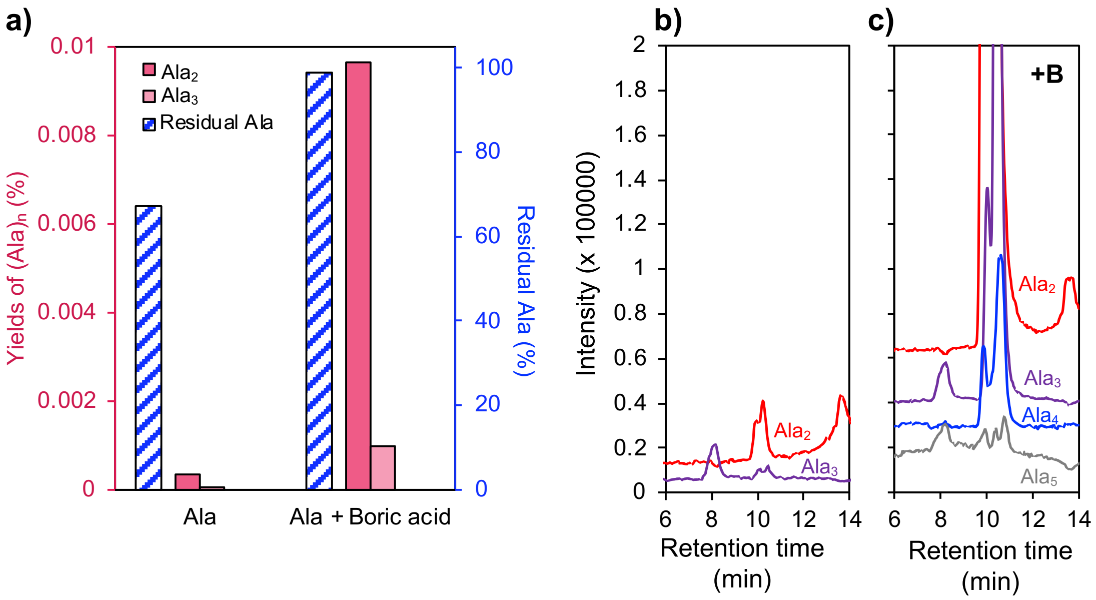

Fig. S8. Yields of short Ala peptides and residual Ala at 130 °C and pH 6 after heating for 250 h. a) Yields of Ala peptides and residual Ala. **b)** Ala peptides detected by LC–MS.

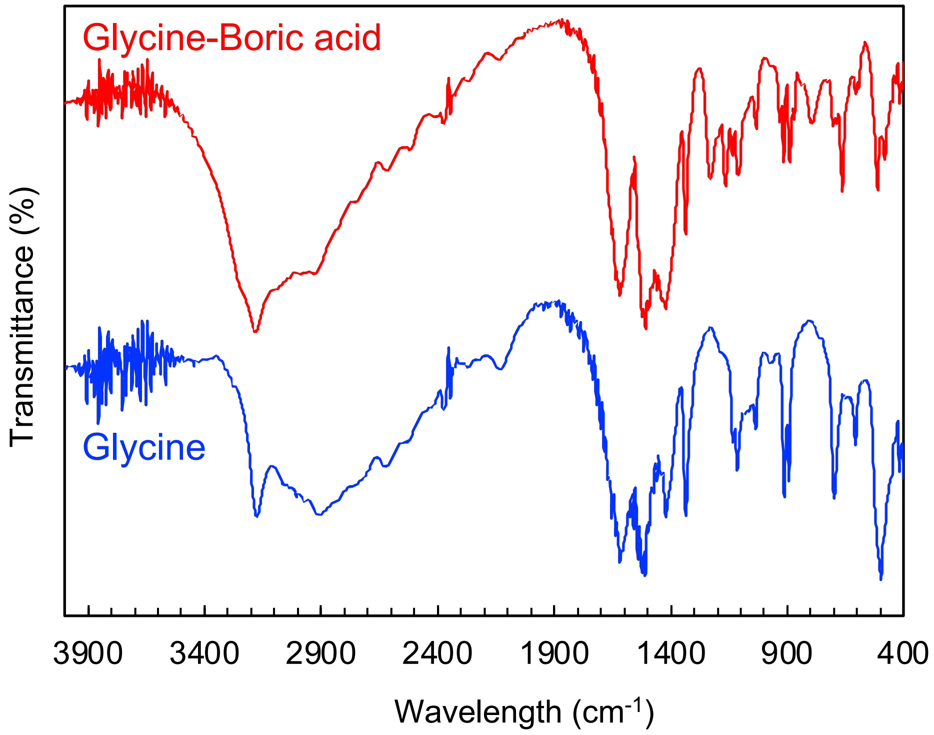

Fig. S9. FT-IR spectra of dried solutions of glycine (pH 6) and the mixture of glycine and equivalent moles of boric acid (pH 5).

Table S1. Mass signals and theoretical monoisotopic masses of Gly peptides.

Oligomers	Composition formulas	Theoretical $[M+Na]^+$	Experimental $[M+Na]^+$	Δ ppm
Gly_5	$C_{10}H_{17}N_5O_6$	326.10710	326.10723	0.39864
Gly_6	$C_{12}H_{20}N_6O_7$	383.12857	383.12846	-0.28711
Gly_7	$C_{14}H_{23}N_7O_8$	440.15003	440.14981	-0.49983
Gly_8	$C_{16}H_{26}N_8O_9$	497.17150	497.17128	-0.44250
Gly_9	$C_{18}H_{29}N_9O_{10}$	554.19296	554.19280	-0.28871
Gly_{10}	$C_{20}H_{32}N_{10}O_{11}$	611.21442	611.21426	-0.26177
Gly_{11}	$C_{22}H_{35}N_{11}O_{12}$	668.23589	668.23583	-0.08979
Gly_{12}	$C_{24}H_{38}N_{12}O_{13}$	725.25735	725.25739	0.05515
Gly_{13}	$C_{26}H_{41}N_{13}O_{14}$	782.27881	782.27894	0.16618
Gly_{14}	$C_{28}H_{44}N_{14}O_{15}$	839.30028	839.30055	0.32170
Gly_{15}	$C_{30}H_{47}N_{15}O_{16}$	896.32174	896.32212	0.42395
Gly_{16}	$C_{32}H_{50}N_{16}O_{17}$	953.34320	953.34372	0.54545
Gly_{17}	$C_{34}H_{53}N_{17}O_{18}$	1010.36467	1010.36530	0.62354
Gly_{18}	$C_{36}H_{56}N_{18}O_{19}$	1067.38613	1067.38681	0.63707
Gly_{19}	$C_{38}H_{59}N_{19}O_{20}$	1124.40760	1124.40849	0.79153
Gly_{20}	$C_{40}H_{62}N_{20}O_{21}$	1181.42906	1181.43028	1.03265
Gly_{21}	$C_{42}H_{65}N_{21}O_{22}$	1238.45052	1238.45138	0.69442
Gly_{22}	$C_{44}H_{68}N_{22}O_{23}$	1295.47199	1295.47226	0.20842
Gly_{23}	$C_{46}H_{71}N_{23}O_{24}$	1352.49345	1352.49454	0.80592
Gly_{24}	$C_{48}H_{74}N_{24}O_{25}$	1409.51491	1409.51618	0.90102
Gly_{25}	$C_{50}H_{77}N_{25}O_{26}$	1466.53638	1466.53769	0.89326
Gly_{26}	$C_{52}H_{80}N_{26}O_{27}$	1523.55784	1523.55832	0.31505
Gly_{27}	$C_{54}H_{83}N_{27}O_{28}$	1580.57931	1580.57936	0.03163
Gly_{28}	$C_{56}H_{86}N_{28}O_{29}$	1637.60077	1637.60237	0.97704
Gly_{29}	$C_{58}H_{89}N_{29}O_{30}$	1694.62223	1694.62400	1.04448
Gly_{30}	$C_{60}H_{92}N_{30}O_{31}$	1751.64370	1751.64521	0.86205
Gly_{31}	$C_{62}H_{95}N_{31}O_{32}$	1808.66516	1808.66497	-0.10505
Gly_{32}	$C_{64}H_{98}N_{32}O_{33}$	1865.68662	1865.68540	-0.65391
Gly_{33}	$C_{66}H_{101}N_{33}O_{34}$	1922.70809	1922.70696	-0.58771
Gly_{34}	$C_{68}H_{104}N_{34}O_{35}$	1979.72955	1979.72830	-0.63140
Gly_{35}	$C_{70}H_{107}N_{35}O_{36}$	2036.75102	2036.75275	0.84939
Gly_{36}	$C_{72}H_{110}N_{36}O_{37}$	2093.77248	2093.77530	1.34685
Gly_{37}	$C_{74}H_{113}N_{37}O_{38}$	2150.79394	2150.79559	0.76716
Gly_{38}	$C_{76}H_{116}N_{38}O_{39}$	2207.81541	2207.81255	-1.29540
Gly_{39}	$C_{78}H_{119}N_{39}O_{40}$	2264.83687	2264.84312	2.75958

Table S2. Mass signals and theoretical monoisotopic masses of the tandem mass fragments from Gly_{15} (Fig. S3).

Experimental [M+Na]+	Theoretical [M+Na]+	Fragment type	Δ ppm
383.12851	383.12857	$[y_6+Na]^+$	-0.15661
422.13928	422.13947	$[b_7+Na]^+$	-0.45009
440.14993	440.15003	$[y_7+Na]^+$	-0.22720
479.16077	479.16093	$[b_8+Na]^+$	-0.33392
497.17132	497.17150	$[y_8+Na]^+$	-0.36205
536.18217	536.18239	$[b_9+Na]^+$	-0.41031
554.19272	554.19296	$[y_9+Na]^+$	-0.43306
593.20360	593.20386	$[b_{10}+Na]^+$	-0.43830
611.21410	611.21442	$[y_{10}+Na]^+$	-0.52355
650.22490	650.22532	$[b_{11}+Na]^+$	-0.64593
668.23546	668.23589	$[y_{11}+Na]^+$	-0.64349
707.24629	707.24679	$[b_{12}+Na]^+$	-0.70697
725.25688	725.25735	$[y_{12}+Na]^+$	-0.64805
764.26769	764.26825	$[b_{13}+Na]^+$	-0.73273
782.27832	782.27881	$[y_{13}+Na]^+$	-0.62638
821.28916	821.28971	$[b_{14}+Na]^+$	-0.66968
839.29967	839.30028	$[y_{14}+Na]^+$	-0.72680
878.31050	878.31118	$[b_{15}+Na]^+$	-0.77421
896.32106	896.32174	$[y_{15}+Na]^+$	-0.75866

Table S3. Amounts and concentrations of additives to adjust the pH of the starting solution.

	pH	additives (μL)			
		1 M HCl	1.7 M NaOHaq	3.4 M NaOHaq	17 M NaOHaq
B-free solution	2	20	0	0	0
	3	6	0	0	0
	6.5	0	0	0	0
	8	0	1.5	0	0
	10	0	0	0	8
B-containg solution	2	20	0	0	0
	3	6	0	0	0
	6	0	0	1	0
	8	0	0	0	3.3
	10	0	0	0	11
